# Augmented GNSS Differential Corrections Minimum Mean Square Error Estimation Sensitivity to Spatial Correlation Modeling Errors

**DOI:** 10.3390/s140610258

**Published:** 2014-06-11

**Authors:** Nazelie Kassabian, Letizia Lo Presti, Francesco Rispoli

**Affiliations:** 1 Department of Electronics and Telecommunications, Politecnico di Torino, Corso Duca degli Abruzzi 24, 10129 Turin, Italy; E-Mail: letizia.lopresti@polito.it; 2 Ansaldo STS, S.p.A, Via Paolo Mantovani 3-5, 16151 Genoa, Italy; E-Mail: francesco.rispoli@ansaldo-sts.com

**Keywords:** GNSS, augmentation system, reference station, differential correction, linear MMSE, correlation distance

## Abstract

Railway signaling is a safety system that has evolved over the last couple of centuries towards autonomous functionality. Recently, great effort is being devoted in this field, towards the use and exploitation of Global Navigation Satellite System (GNSS) signals and GNSS augmentation systems in view of lower railway track equipments and maintenance costs, that is a priority to sustain the investments for modernizing the local and regional lines most of which lack automatic train protection systems and are still manually operated. The objective of this paper is to assess the sensitivity of the Linear Minimum Mean Square Error (LMMSE) algorithm to modeling errors in the spatial correlation function that characterizes true pseudorange Differential Corrections (DCs). This study is inspired by the railway application; however, it applies to all transportation systems, including the road sector, that need to be complemented by an augmentation system in order to deliver accurate and reliable positioning with integrity specifications. A vector of noisy pseudorange DC measurements are simulated, assuming a Gauss-Markov model with a decay rate parameter inversely proportional to the correlation distance that exists between two points of a certain environment. The LMMSE algorithm is applied on this vector to estimate the true DC, and the estimation error is compared to the noise added during simulation. The results show that for large enough correlation distance to Reference Stations (RSs) distance separation ratio values, the LMMSE brings considerable advantage in terms of estimation error accuracy and precision. Conversely, the LMMSE algorithm may deteriorate the quality of the DC measurements whenever the ratio falls below a certain threshold.

## 1. Introduction

Railway signaling, a baseline safety system to control the movement of trains, has emerged years after the dawn of the first steam engined powered locomotives. The motivation behind this safety system for railways is the need of a much greater distance to stop than on road due to tenuous contact between steel wheel and steel rail. In the early days of railways, a simple time interval system was the rule of thumb to avoid train collisions due to the absence of any communication or signaling between trains. In fact, trains used to wait for 10 min, known as “headway” before being given the green light to depart from the station. This obviously decreased line capacity. Railway signaling has come a long way ever since, evolving to fixed mechanical signaling and later in the beginning of the twentieth century, to automatic signaling by means of electronic track circuits and transponders also known as balise. These transponders give the exact position of the train as it passes through it, and are named Eurobalise if they comply with the European Rail Traffic Management System (ERTMS)/European Train Control System (ETCS) specification.

Nowadays, satellite localization and navigation systems coupled with telecommunication systems are considered to be an efficient solution to significantly reduce costs related to railway track equipments and their maintenance, as well as to increase line capacity and provide more reliable railway services [1]. It is important however to verify that Global Navigation Satellite System (GNSS) can offer the safety requirements imposed by the ERTMS/ETCS specification. It is shown in [2] that the Galileo safety of life service, which was mainly designed for aeronautical operations, but is also applicable to railway applications with a different safety philosophy, can be assessed through quality attributes specific to railway signaling, that is Reliability, Availability, Maintainability and Safety (RAMS).

**Figure 1.**
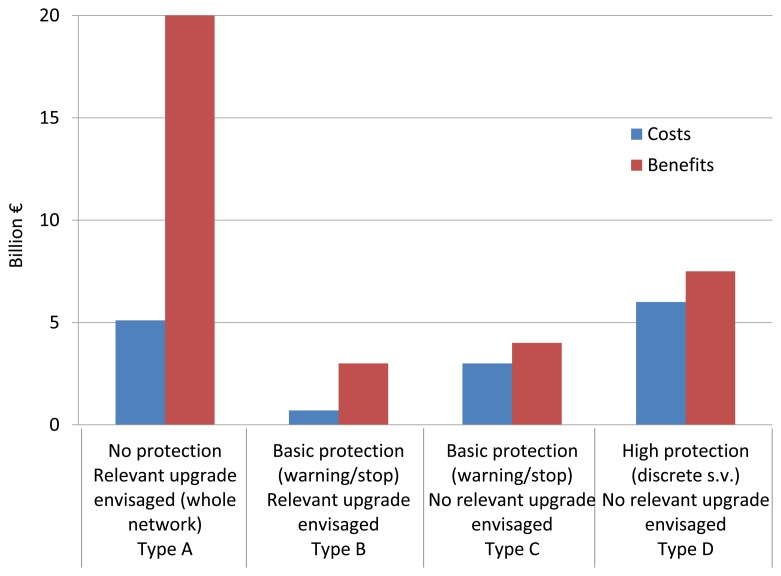
Comparison of costs and benefits (billion of €) of the project for the European regional network (selected countries) by Type of context (Data from [3]).

The ETCS specification, is a de-facto standard widely accepted in the world for signaling and speed-control applications. In the Memorandum of Understanding signed in 2012 between the European Commission, European Railway Agency (ERA) and the rail stakeholders it is foreseen that the next generation of ERTMS will be upgraded with additional functionalities including GNSS technology. In Europe, the ERTMS solution with satellite tracking is motivated by the needs of modernization of local and regional lines, which represent about 50 percent of all lines and carry a total number of passengers twenty times higher than the number of passengers transported by aircraft. The adoption of the GNSS represents an unprecedented innovation backed by the important economical benefits and the contribution to improve the safety especially on the local and regional lines where no protection systems are employed and human errors may be fatal, as in Figure 1. The main challenge is the elimination of track-side equipments along the lines with the safe localization of the train by the GNSS that will imply the use of augmentation networks with proper algorithms to assure the correctness and liability of the train position.

**Figure 2.**
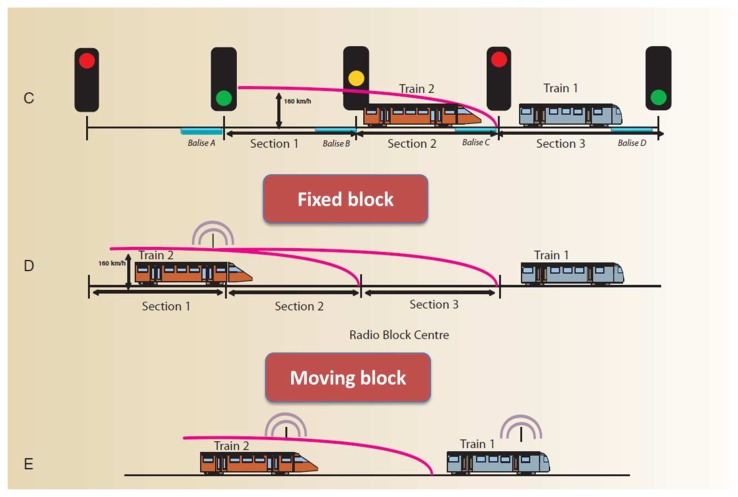
ERTMS standard configurations.

In fact, given that the positioning accuracy of standalone GNSSs is of the order of a few meters, it is important to make use of augmentation systems and differential techniques to drive the accuracy down as low as a couple of meters and maximize continuity and integrity [4,5]. Aviation applications do so by making use of Ground Based Augmentation Systems (GBASs) such as Local Area Augmentation Systems (LAASs) and Space Based Augmentation Systems (SBASs) such as the Wide Area Augmentation System (WAAS) and the European Geostationary Navigation Overlay System (EGNOS). These SBASs employ geostationary satellites and a network of terrestrial receivers scattered over large distances such as continents and countries. In this way, a SBAS mitigates a wide variety of GNSS error sources like ionosphere, troposphere and satellite clock errors [6]. In a similar way, GBASs and/or SBASs can serve the railway signaling and protection systems to increase accuracy, continuity and integrity of position and velocity GNSS measurements. For railway applications, the integrity of the elaborated train position is crucial to meet the safety requirements. In addition to integrity, the user differential corrections generated by the set of Reference Station (RS) play an important role to assure that the train can be stopped behind critical points and in some cases, respect the railway traffic lights or signals as shown in Figure 2. Normally, these signals are placed at the beginning and end of each block, which is a section of the track between two fixed points, and are preceded by transponders to detect the train position as it passes nearby. This is basically a fixed block setting where train positions are only known at the balise locations, and therefore display a constant worst-case headway based on a discontinuous location monitoring. The moving block setting on the other hand, does not make use of fixed points, but rather is based on a continuous, precise and timely information on the location of the front and rear of each train. This in turn increases line capacity. GNSS is an enabling technology of such a setting, and can be used together with other types of sensors to maximize continuity and integrity.

**Figure 3.**
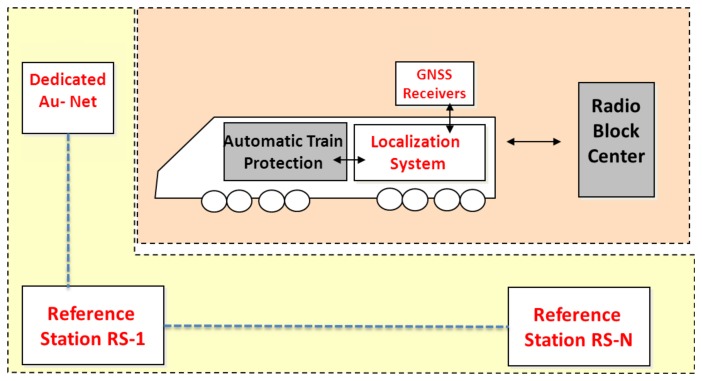
Satellite-based train control system (3InSat project).

This paper explores the differentially corrected pseudorange accuracy that can be reached by harnessing the existence of several communicating RSs in the vicinity of railway tracks. In fact, the complexity and cost of the augmentation network depends on the number of reference stations. Although this study is inspired by the railway application, it is not limited to it and the problem that is dealt with is of great concern to all transportation system applications that need an augmentation system in order to deliver more accurate and reliable positioning with integrity specifications. A handful of such examples can be found in the road sector for safety critical and commercial services, such as management of hazardous goods transport and electronic toll collection systems [7,8]. Moreover, a dedicated augmented network is proposed in [1,9], to provide augmentation data for railway applications, making reference to the ERTMS system supported by the GNSS localization. Figure 3 shows the general architecture of the ERTMS system with the GNSS solution. The dedicated augmentation network is equipped with ranging and integrity monitoring RS, communication base stations, location determination systems, and a track area location determination system safety server which processes the outputs sent by the RSs [1,9]. In this paper, a general study of the minimum distance separation between RSs is carried out indirectly by considering different values of the correlation distance, the distance that separates two points characterized by correlated error sources. This is due to the inverse proportionality relationship that exists between the correlation distance and the distance that separates RSs.

In conclusion, the Linear Minimum Mean Square Error (LMMSE) algorithm is a classical estimation method widely used in augmented GNSS applications [10,11]. It is based on spatial correlation models, that are often defined for each error source, such as ionosphere, troposphere and satellite ephemeris. The contribution of this paper resides in assessing the sensitivity and robustness of the LMMSE algorithm to modeling errors in a typical railway scenario. The methodology of this study can be applied to any augmentation based system, as long as the spatial correlation models take into account the geometry of the application and the dedicated augmentation network. In the following, the spatial correlation model is considered to encompass all error sources. The paper is organized as follows. Section 2 introduces the pseudorange Differential Correction (DC) expression generated by these RSs together with its second order statistics model. In addition, it introduces the linear LMMSE algorithm to estimate the true DC from the vector of noisy measurements relative to these RSs. Section 3 on the other hand, describes the pseudorange DC simulation process for a number of correlation distance values, and shows the sensitivity of the estimation algorithm to different modeling errors.

## 2. LMMSE Estimator within GNSS Augmentation Systems

Similar to aviation applications of local area and wide area differential GNSS, the railway scenario can take advantage of well surveyed GNSS reference stations to generate differential corrections for pseudorange measurements obtained by a standard GNSS receiver. In the vicinity of railway lines, every GNSS equipped RS is able to derive pseudorange measurements between itself and each individual Space Vehicle (SV) in view. On the other hand, these RSs have well known positions as part of an augmentation system infrastructure. Differential corrections are hence generated, which are fairly accurate but affected by noise.

### 2.1. Pseudorange Measurement Error Modeling

The pseudorange DC or measurement error for each satellite *i* and RS *j* at every epoch *n* can be written as [12]:

(1) $$\eta_{i,j}=c\left(\delta t_{j}-\delta t_{i}\right)+E_{i,j}+I_{i,j}+T_{i,j}+\epsilon_{i,j}$$

where c is the speed of light, *δt_j_* and *δt_i_* are the reference station and satellite clock bias from a standard time reference as GPS Time (GPST), *E_i,j_* is the ephemeris error, *I_i,j_* is the ionosphere delay bias, *T_i,j_* is the troposphere delay bias, and *ϵ_i,j_* is the error in unmodeled effects, modeling errors, and measurement errors. The pseudorange measurement error is the result of several independent error sources, which are individually modeled by an overbounding zero mean normal distribution characterized by a standard deviation *σ_CE_* for satellite clock and ephemeris errors, *σ_I_* for ionosphere errors, *σ_T_* for troposphere errors and *σ_w_* for unmodeled noisy errors. Due to the noisy term *ϵ_i,j_* in the expression of the pseudorange DC, it is possible to separate the expression in two terms, that is

(2) $$\eta_{i,j}=\xi_{i,j}+\epsilon_{i,j}$$

where *ξ_i,j_* = *cδt_j_* + *E_i_*_,_*_j_* + *I_i,j_* + *T_i,j_* is the true total correction to be estimated. It is modeled by a normal distribution with zero mean and a variance of $\sigma_{\xi }^{2}=\sigma_{CE}^{2}+\sigma_{I}^{2}+\sigma_{T}^{2}$. Considering a number of RSs of known position, performing pseudorange measurements along the railway line, at each epoch *n*, and for each satellite *i*, the sequence of *ξ_i,j_* can be written in vector form as $\xi =\left[\xi_{i,1,}\xi_{i,2,}\cdots ,\xi_{i,N_{RS}}\right]$, where *N_RS_* is the number of RSs (in ***ξ*** the symbol *i* is omitted to simplify the notations). To take into account the spatial correlation among *ξ_i,j_* and their random nature, ***ξ*** is modeled as a stochastic vector defined by a Probability Density Function (PDF) as ***ξ*** ∼ 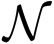
(***μ_ξ_***,***R_ξ_***). The modeling and measurement errors which make up the noisy term are independent of time and space and other RSs. Therefore, the vector of noisy terms relative to each RS is modeled by a zero mean jointly Gaussian random process with uncorrelated components in time and a diagonal covariance matrix in space, defined by a PDF as ***ϵ*** ∼ 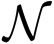
(**0**,***R_w_***) and ${\text{R}}_{w}=\sigma_{w}^{2}\text{I}$, where ***I*** is the identity diagonal matrix. The resulting pseudorange DC is then represented by vector ***η***.

### 2.2. Estimating True Total DC

The true total DC vector ***ξ***, characterized by a covariance matrix ***R_ξ_***, can be estimated using a classical LMMSE algorithm, as in [13]. It is worth noting that a similar approach is applied in [11], where the LMMSE algorithm is applied to estimate vertical ionospheric delays at the considered user position starting from vertical ionospheric delays measured at various WAAS RSs. In this paper, the user is not taken into consideration, but only the network of RSs. This study intends to explore the quality of the true DC estimates that can be reached by harnessing the pseudorange DC generated by this network of RSs with fixed surveyed positions and a-priori knowledge of their mutual second order statistics.

In fact, the approach in [11] makes a key assumption, mainly that the correlation of the vertical ionospheric delay between two points of the ionospheric grid depends only on the distance between these two points. The same is true for satellite ephemeris errors and propagation errors such as troposphere delay [6]. By noting that the different error sources acting on the true total DC are independent, the corresponding total covariance matrix can be written as the sum of the individual covariance matrices:

(3) $${\text{R}}_{\xi }=\sigma_{CE}^{2}\exp\left(-\text{x}/D_{E}\right)+\sigma_{I}^{2}\exp\left(-\text{x}/D_{I}\right)+\sigma_{I}^{2}\exp\left(-\text{x}/D_{T}\right)$$

where *D_E_*, *D_I_* and *D_T_* are the correlation distances relative to the correlation functions characterizing the satellite ephemeris, ionosphere and troposphere corrections at different RSs respectively. The satellite clock error is the same for all users and thus is discarded in the covariance matrix. The spatial decorrelation value or the ionosphere gradient, is reported to be in the range of 1-4 mm/km in active but not stormy ionosphere conditions where it can go up to 400 mm/km [6]. The ionosphere correlation distance, which is the reciprocal of the spatial decorrelation, can thus be deduced to be in the range of 250-1000 km in normal conditions and go down as low as 2.5 km during large geomagnetic storms. Likewise, the ephemeris gradient is lower than 0.5 mm/km while the worst-case tropospheric decorrelation value is reported to be around 5 mm/km in [6].

**Figure 4.**
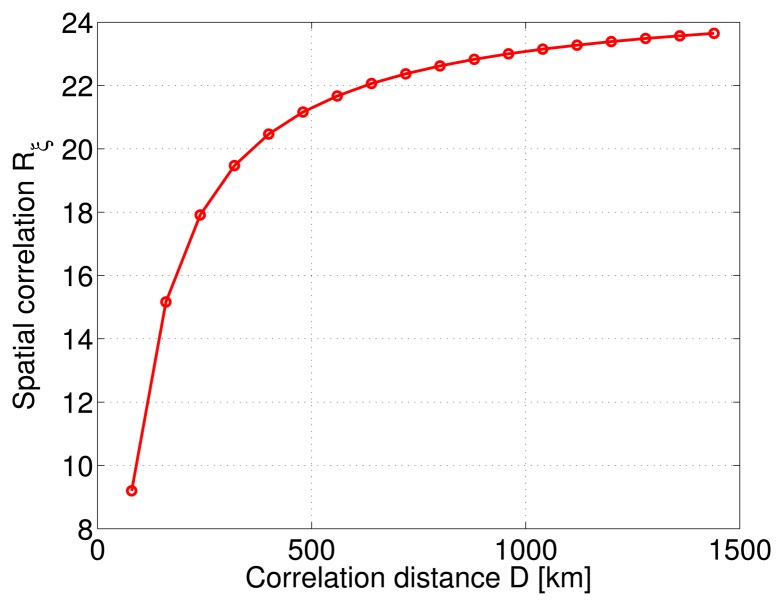
Spatial correlation as a function of correlation distance with a fixed distance separation.

Going back to Equation (2), the LMMSE estimate of a vector of random variables buried in noise is [13]:

(4) $$\overset{\text{\textasciicircum{}}}{\xi }\;=\;\mu_{\xi }+{\text{R}}_{\xi }{\left({\text{R}}_{\xi }+{\text{R}}_{\text{w}}\right)}^{-1}\left(\eta -\mu_{\eta }\right)$$

where it is assumed that the modeling errors and the true DC are uncorrelated and independent Gaussian Random Variable (RV). Given that the outcome of the estimation algorithm is most adversely affected by the narrowest correlation function, the sum of the three correlation functions can be approximated to be equal to one correlation function considering the lowest correlation distance, and with a variance coefficient equal to the sum of the three variances. This motivates the fact that it is commonplace to model the true total DC covariance matrix by a single exponential function characterized by a constant correlation distance *D* and a pseudorange DC standard deviation *σ* [10,11] as:

(5) $${\text{R}}_{\xi }=\sigma^{2}\exp\left(-\text{x}/D\right)$$

where ***x*** is a 2-Dimensional matrix considering all distance combinations between RSs spread along the rectilinear railroad track. The behavior of this spatial correlation for a fixed distance separation and a variable correlation distance is shown in Figure 4. It can be concluded that, for a fixed distance separation, the spatial correlation value increases with the correlation distance following a horizontal asymptote. However, the LMMSE estimation method can also be applied considering the sum of individual covariance matrices as shown in Equation (3). In fact, in this case, the parametric analysis involves six modeling error parameters, *i.e.*, the three error sources ephemeris, ionosphere, troposphere, each of which modeled by two parameters, the variance and the correlation distance. This case will be further discussed in the following section.

### 2.3. Estimation Algorithm Considerations

In order to use Equation (4), the following vector and two matrices have to be known:

- ***μ_ξ_*** approaches ***μ_η_*** after a certain initialization time where a considerable amount of measurements is performed to assume that the average operation drives the contribution of ***ϵ*** to zero.
- ***R_w_*** depends on the quality of the GNSS receiver and is intrinsic to it. In fact, $\sigma_{w}^{2}$ can be estimated offline and is considered to be a known vector.
- ***R_ξ_*** is the most critical estimate, and is defined by two variables, the pseudorange DC standard deviation *σ* and the correlation distance *D* as seen from Equations (3) and (5). These two variables have to be estimated by surveying campaigns beforehand (two methods are described in [10]) and depend on the environmental conditions which can vary in time. Therefore, it is important to assess the situation when the modeling parameters diverge from true values due to several reasons, such as geometric changes and severe ionospheric storms or sudden atmospheric changes [10]. The objective of this paper is thus to analyze the sensitivity of the LMMSE algorithm to variations of *σ* and *D*.

## 3. Simulation Results

Monte-Carlo simulations are carried out in order to assess the performance of the LMMSE algorithm in estimating the true total DC from a set of measurements available at RSs and testing its sensitivity to modeling errors. It is assumed that five RSs are distributed along a railway track of 400 km, *i.e.*, separated by 80 km. In [9], the same number of RSs are distributed over a railway track of 300 km and considered to be separated by a distance less than the decorrelation distance. In the following, three cases will be considered: the case where the true total DC is simulated and modeled according to a correlation function that is a single exponential function as in Equation (5), or the sum of multiple exponential functions with different correlation distance as in Equation (3) or the sum of multiple exponential functions with the same correlation distance.

### 3.1. Simulating the True Total DC

The true total DC vector ***ξ*** is simulated considering a first order Gauss-Markov process with a decay rate parameter of 1/*D* and process variance equal to the sum of the individual variances $\sigma_{CE}^{2}=0.85^{2}\;m^{2}$, $\sigma_{I}^{2}=9^{2}\;m^{2}$ and $\sigma_{T}^{2}=1\;m^{2}$ taken to be representative of worst-case values [7]. This is equivalent to generating different values of the error sources at each RS. Moreover, the simulated vector as such yields the exponential decay rate function that characterizes the true total DC covariance matrix as in Equation (5). The mean of the simulated vector as such is zero, however the pseudorange DC for a standard user is around 3-10 m, reflecting the ionosphere, troposphere, unmodeled effects and modeling/measurement errors. Therefore, a constant error of *σ_ξ_* m is manually added to the simulated DC vector to take into account biases such as satellite ephemeris and clock errors, as well as propagation errors (ionosphere and troposphere), while keeping the structure of the correlation matrix ***R_ξ_***. It is worth noting that adding a bias to the simulated process vector ***ξ*** as such maintains the diversity of the errors at each RS.

Alternatively, the true total DC vector ***ξ*** is simulated as a sum of three first order Gauss-Markov processes having the same decay rate parameter value of 1/*D* but different process variances $\sigma_{CE}^{2}$, $\sigma_{I}^{2}$ and $\sigma_{T}^{2}$. In this case, ***ξ*** can be seen as the output of the sum of three white noise signals ***W_i_*** ∼ 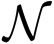
(**0**,***σ_w_i__***) passed through a low pass filter **H**(**z**):

(6) $$H(z)=\frac{d\sqrt{2\alpha }}{1+\alpha d-z^{-1}}=\frac{a}{1-bz^{-1}}$$

with $a=\frac{d\sqrt{2\alpha }}{1+\alpha d}$, $b=\frac{1}{1+\alpha d}$ *α* =1/*D*, *d* = 80 km which represents the RS distance separation and ***σ_w_i__*** represent the aforementioned variances relative to the satellite clock and ephemeris error, the ionosphere and troposphere errors. The sum of the output of these three filters can then be written as:

(7) $$\xi [n]=\sum_{l=-\infty }^{\infty }h[l]\sum_{i=1}^{3}w_{i}\left[n-l\right]=\sum_{l=-\infty }^{\infty }h[l]w\left[n-l\right]$$

with *h*[*n*] = *ab^n^* is the Z inverse transform of *H*(*z*) and *w*[*n*] a white noise signal with a normal distribution $N\left(0,\sqrt{\sum \sigma_{w_{i}}^{2}}\right)$. This proves that the true total DC vector generated as such is still a Gauss-Markov process, with the same decay rate parameter and variance $\sigma_{\xi }^{2}=\sqrt{\sum \sigma_{w_{i}}^{2}}$.

On the other hand, as previously mentioned, the vector of noise terms ***ϵ*** is modeled as a zero mean jointly Gaussian random process with a diagonal covariance matrix defined by ${\text{R}}_{w}=\sigma_{w}^{2}\text{I}$ with $\sigma_{w}^{2}=\sigma_{m}^{2}+\sigma_{r}^{2}$ and *σ_m_* = 1.5 m and *σ_r_* = 0.35 m as approximate maximum 1-Sigma error values on code phase measurements. The aforementioned error values *σ_m_* and *σ_r_* are due to multipath and receiver noise respectively considering a stand-alone user (not using Differential GNSS (DGNSS) corrections) [6]. It is worth noting that such values are independent between user and reference receivers and correspond to clean or moderate reflecting source conditions around user and reference receivers. Moreover, it is assumed that a default level of carrier smoothing is present. Finally, a vector of correlation distance ***D*** values and variance values ***σ*^2^** is considered in generating the true total DC.

### 3.2. Estimating the True Total DC

Assuming that one has a simultaneous access to the corrections generated at each RSs, the true total DC vector ***ξ*** is estimated using the LMMSE as in Equation (4). It is assumed that ***μ_ξ_*** = ***μ_η_*** as previously mentioned, and that a relatively good estimate of the true total DC covariance matrix ***R̂_ξ_*** modeled as a single exponential function is obtained. However, it is essential to assess the impact of the spatial correlation matrix modeling errors on the true total DC estimation quality. For that end, a range of modeling errors Δ***D*** = 0 to 2000 km are added to the vector ***D*** which holds the true correlation distance values considered in simulating the true total DC vector. Moreover, a range of true total DC variance modeling errors Δ***σ*^2^** = 0 to 25 m^2^ is added to the true value ***σ*^2^** which in these simulations coincides with $\sigma_{\xi }^{2}$. The results presented herein are relative to a simulated true total DC.

**Figure 5.**
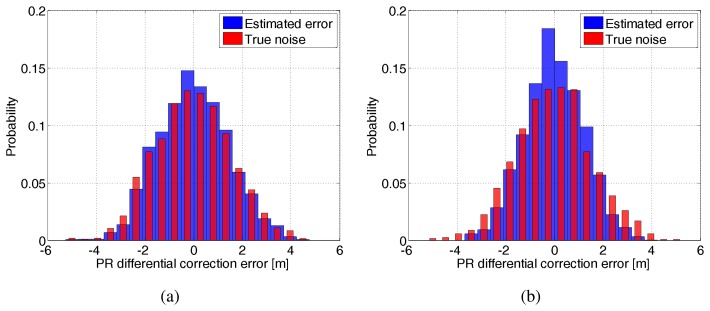
Superimposed histogram of estimation error and true error distributions for a single combination of modeling errors (**a**) Δ*D* = 200 km on top of *D* = 100 km and (**b**) Δ*D* = 1800 km on top of *D* = 900 km.

In order to assess the LMMSE estimation quality, a Monte-Carlo simulation of 500 runs has been carried out: the true total DC vector is simulated 500 times, the corresponding LMMSE estimates are generated, and the estimation error is computed. Figure 5 shows on the same plot, the histogram of both the estimation error distribution and the true noise distribution for two individual cases: (a) a true distance correlation of 100 km and a modeling error Δ*D* = 200 km in Figure 5a and a corresponding (b) 900 km and a modeling error of 1800 km in Figure 5b. Both the estimation error and true noise refer to all RSs which are separated between themselves by a constant distance *d* = 80 km. It can be concluded from these two histograms that the LMMSE estimation yields no bias errors and generally reduces the noise affecting the true total DC, as the probability of estimation error lying around the 0 bin is higher than that corresponding to the true error.

**Figure 6.**
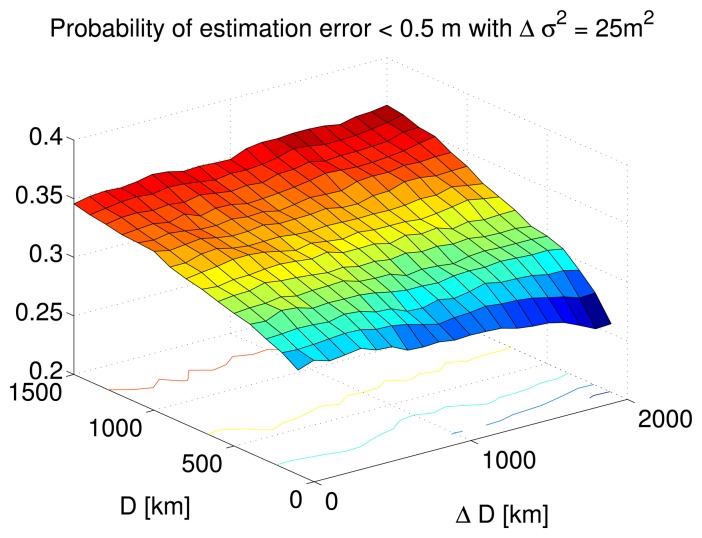
Probability of estimation error lying below half a meter for different correlation distance values and corresponding modeling errors.

Furthermore, this probability is slightly higher for a larger correlation distance value of 900 km as seen in Figure 5b compared to 100 km Figure 5a. This is further demonstrated in Figure 6 where the probability of the estimation error lying below half a meter is plotted with respect to various combinations of correlation distance and corresponding modeling error. The DC variance modeling error is taken to be 25 *m*^2^. It can be seen that this probability is higher as the correlation distance increases irrespective of the modeling error magnitude.

**Figure 7.**
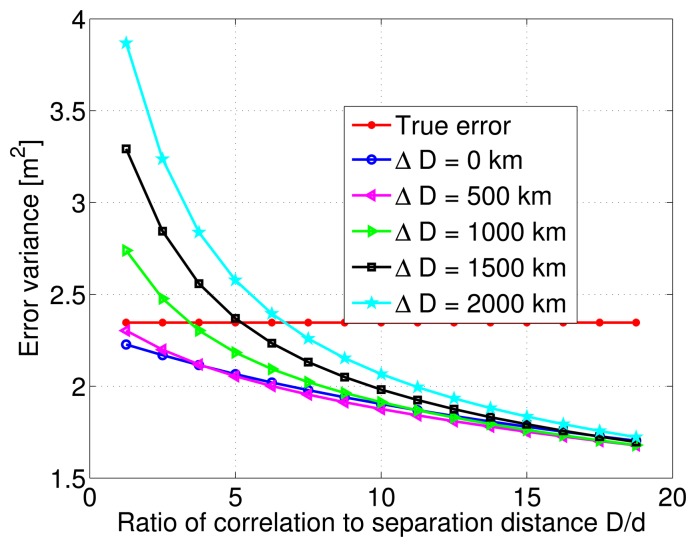
Estimation error variance corresponding to a single Gauss-Markov process and different Δ*D* modeling errors.

After this initial test on the estimation quality, the variance of the estimation error is numerically computed from the Monte-Carlo simulations, and is plotted as a function of the ratio between correlation distance values and a constant distance separation of 80 km. Multiple curves are overlayed on the same figure to indicate the behavior of the various modeling errors. Figure 7 shows that although the estimation error variance decreases with increasing ratio of correlation to separation distance values, it does not change much after a certain ratio value. This is true for all cases of correlation distance modeling errors. The true error or noise is also plotted, and it can be seen that for correlation distance modeling errors ranging from 0 to 2000 km, the estimation error variance is always lower than the true error variance as long as the ratio *D*/*d* is greater than 5. The important and unusual conclusion to draw here, is that within the range of the considered modeling errors, the LMMSE algorithm might even deteriorate the quality of the pseudorange DC estimate. In fact, the estimation error variance considerably increases as the ratio *D*/*d* drops below 5, and modeling errors rise above 500 km.

**Figure 8.**
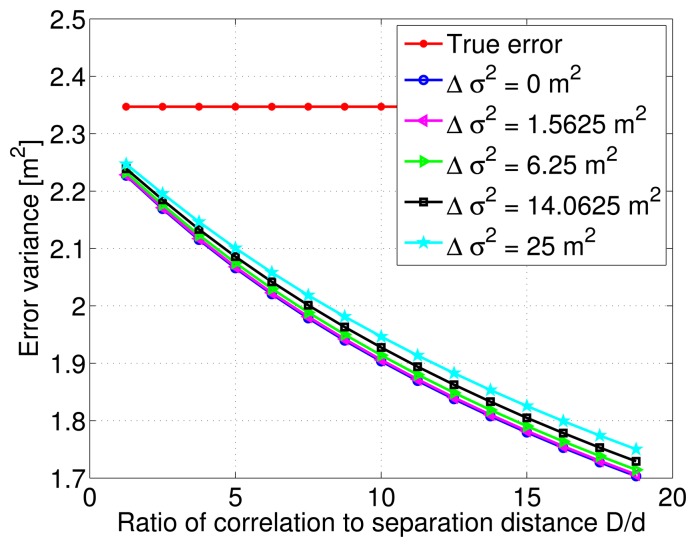
Estimation error variance corresponding to a single Gauss-Markov process and different Δ*σ*^2^ modeling errors.

In addition, Figure 8 shows the decreasing behavior of the estimation error variance with increasing correlation distance and ratio *D*/*d*. The various curves indicate different variance modeling error values relative to the true total DC. There is a slight vertical shift between these curves, and as noticed previously, the estimation error variance decreases even more slowly with large correlation distance values.

### 3.3. Further Analysis of True Total DC Estimation

**Figure 9.**
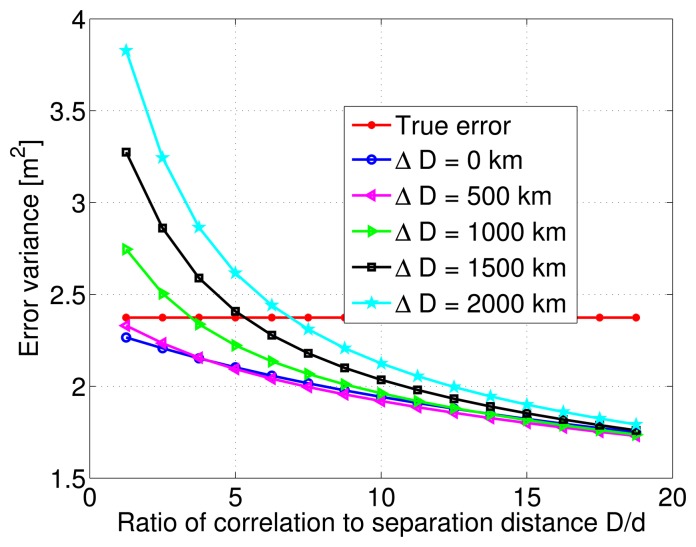
Estimation error variance corresponding to a sum of three Gauss-Markov processes and different Δ*D* modeling errors.

**Figure 10.**
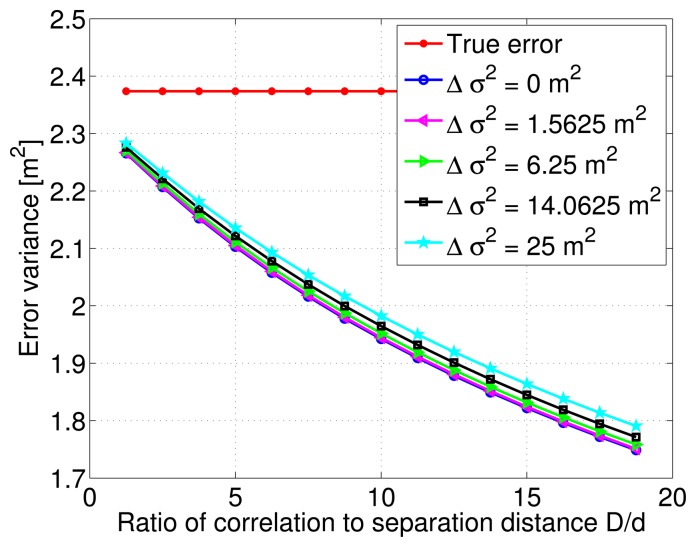
Estimation error variance corresponding to a sum of three Gauss-Markov processes and different Δ*σ*^2^ modeling errors.

This section presents results of estimating true total DC with the alternative method of summing three Gauss-Markov processes with the same or different correlation distance values for the different error sources. Similar results as presented in the previous section, are achieved in case the correlation distance is assumed the same for satellite ephemeris, ionosphere and troposphere error correlation functions. The focus herein is the case where the true total DC is simulated as the sum of three Gauss-Markov processes with different correlation distance values:

(8) $$\xi [n]=\sum_{l=-\infty }^{\infty }\sum_{i=1}^{3}h_{i}[l]w_{i}\left[n-l\right]=\sum_{l=-\infty }^{\infty }\sum_{i=1}^{3}a_{i}b_{i}^{l}w_{i}\left[n-l\right]$$

with $a_{i}=\frac{d\sqrt{2\alpha_{i}}}{1+\alpha_{i}d}$, $b_{i}=\frac{1}{1+\alpha_{i}d}$, and *α_i_* = 1/*D_i_*. In this case, the true total DC covariance matrix is the sum of three covariance matrices with different correlation distance values as in Equation (4). As concluded from the previous section, the higher the correlation distance for a fixed value of the distance separating RSs, the lower is the impact of the modeling errors. Therefore, at a first thought, only the variance and correlation distance relative to the most narrow correlation function that is characterized by the lowest correlation distance ought to be taken into consideration. However, the ionosphere error is characterized by the largest variance as compared to the other error sources and so determines the correlation value in Equation (3). Therefore, only the variance and correlation distance of the ionosphere error correlation matrix ought to be considered herein. It is thus safe to say that this analysis is based on varying two parameters instead of the six aforementioned parameters. The estimation error variance plotted in Figures 9 and 10, is computed numerically as described in the previous section. As expected, Figures 9 and 10 are very similar to Figures 7 and 8 because the impact of the troposphere, clock and ephemeris error correlation matrices on the total DC estimation is marginal with respect to the ionosphere error correlation matrix. The same conclusion holds, mainly that as the correlation distance and variance modeling errors increase, the performance of the LMMSE algorithm equals or is better than the true error variance for higher values of the correlation distance.

## 4. Conclusions

The adoption of the GNSS for railway signaling applications requires a safe and liable determination of train position and this paper has analyzed one of the core algorithms of the augmentation network. A study of the sensitivity of the LMMSE algorithm to modeling errors regarding the a priori spatial correlation of the true total pseudorange DC is undertaken. The impact of the correlation distance modeling errors decrease with the increase of the true correlation distance to RS distance separation ratio, and converges to an asymptote beyond a certain threshold. On the other hand, noise variance modeling errors have a low impact on the estimation error variance. It is shown that for fairly high correlation distance to RSs distance separation ratio values, and for a wide range of modeling errors, the LMMSE algorithm further decreases the noise inherent in pseudorange DC measurements generated at each RS for every satellite. Conversely, for very low ratio values, and for some correlation distance modeling errors, the LMMSE algorithm increases the noise inherent in pseudorange DC measurements. In this sense, this paper has presented a methodology to assess the required distance between RSs that make up a GNSS augmentation system given a specific correlation distance that characterizes a certain environment. Furthermore, this impacts on the costs for deploying and maintaining the augmentation network.
